# Mental illness stigma and treatment-seeking willingness in Japan: a national survey study

**DOI:** 10.1186/s12939-026-03007-6

**Published:** 2026-10-01

**Authors:** Andrew M. Subica, Shinichi Nagata

**Affiliations:** 1 Department of Social Medicine, Population and Public Health, University of California, Riverside School of Medicine, 900 University Ave, Riverside, CA 92521 USA; 2 Institute of Health and Sport Sciences, University of Tsukuba, 1-1-1 Tennoudai, Tsukuba, 305-8577 Japan

**Keywords:** Japan, Public stigma, Mental health treatment-seeking willingness, Mattering

## Abstract

**Background:**

Japanese adults report high mental illness stigma and low treatment use, yet the specific public stigma beliefs and attitudes associated with Japanese treatment seeking remain unclear. This study had three aims: (1) describe public stigma in a national Japanese sample and compare these patterns descriptively with U.S. public stigma, (2) examine which Japanese public stigma components independently predicted Japanese treatment-seeking willingness, and (3) examine the effects of the potential facilitators of mattering and prior treatment experience on Japanese treatment-seeking willingness.

**Methods:**

Data were collected from a geographically representative national online sample of 1,012 Japanese adults, assessing treatment-seeking willingness and vignette-based measures of public stigma involving perceived seriousness and treatability, causal attributions, desired social distance, and perceived dangerousness. Japanese participants were compared with U.S. adults from the 2018 U.S. General Social Survey. Linear regression examined public stigma predictors and potential facilitators (e.g., mattering) of Japanese treatment-seeking willingness.

**Results:**

Only 54% of Japanese participants reported treatment-seeking willingness, with low perceived treatment need. In descriptive cross-national comparisons, Japanese participants reported significantly lower rates of perceived mental disorder seriousness, lower beliefs in treatment effectiveness/recovery, and fewer neurobiological attributions, with higher desired social distance but lower perceived dangerousness than U.S. adults. Within the Japanese sample, believing treatment would improve mental disorders, greater mattering, and prior treatment experience independently predicted greater treatment-seeking willingness, while believing disorders would improve without treatment and greater desired social distance predicted lower willingness.

**Conclusions:**

Findings revealed a pattern of Japanese public stigma marked by high social distancing and treatment/recovery pessimism that may contribute to low treatment-seeking willingness and unmet mental health need. Positively, mattering emerged as a possible treatment-seeking facilitator. Public stigma and mattering may therefore represent potentially modifiable treatment-seeking barriers and facilitators, respectively, with implications for supporting mental health equity in Japan and addressing Japan’s treatment gap.

## Background

Japan is facing a considerable mental health crisis caused by concerning rates of depression, anxiety, and severe distress [1, 2] combined with low rates of mental health treatment seeking [3]. Japanese adults use mental health treatment at less than half the rate of other developed nations [3] with national data revealing that 88% of Japanese adults with mental disorders do not utilize past-year treatment [4]. This mismatch between Japan’s high mental health need but low treatment use has created a public health problem with national impact [1]. This treatment gap also represents an important mental health equity concern, as adults with mental disorders are likely to experience disproportionate mental health burden along with potential social stigma in Japan while remaining disconnected from available treatment options.

Globally, the leading barrier to mental health treatment seeking is mental illness stigma [5, 6]: the process by which individuals with mental disorders are labeled, stereotyped, and excluded from society by the general public [7]. Because stigma threatens individuals with mental disorders with prejudice, discrimination, ostracization, and loss of social status, income, and economic/vocational opportunities [8, 9], many individuals with mental disorders often avoid seeking treatment to avert these negative consequences [10].

While contemporary stigma frameworks primarily distinguish between public stigma and self-stigma—with structural stigma representing a third less frequently examined stigma type—public stigma in particular appears to play an important role in suppressing treatment seeking on a population level [11–13]. Public stigma refers to the negative stereotypes, prejudicial attitudes, and discriminatory behaviors directed by the general public toward individuals with mental disorders [14, 15], whereas self-stigma is defined as the process by which individuals with mental disorders personally endorse, accept, and apply public stigma stereotypes and prejudices to themselves, leading to feelings of shame, diminished self-worth, and reduced self-efficacy [16]. In extant research, public stigma is frequently operationalized through measures of moralizing causal attributions (e.g., “weak character”), dangerousness stereotypes, and desired social distance from individuals with mental disorders, consistent with how these constructs have been measured in established stigma literature for over two decades [15, 17].

Although both public stigma and self-stigma have been associated with decreased treatment seeking [14, 16], this study specifically examined public stigma and its treatment-seeking impact—in the form of Japanese stigmatizing beliefs, attitudes, and treatment/recovery beliefs toward mental disorders—for three reasons. First, cross-national research has hinted that mental illness stigma is stronger in Japan than in other developed nations [18, 19], yet the specific public stigma beliefs and attitudes that may suppress Japanese treatment seeking remain poorly understood; hindering efforts to improve treatment seeking to reduce Japan’s high unmet need [4]. For example, previous research has found that Japanese adults report greater social distancing from and limited contact with individuals with mental disorders as well as lower mental health literacy [20, 21], and lower expectations for mental illness recovery versus adults in other nations [22, 23]. However, prior research studying stigma’s effects on treatment seeking in Japan has yielded inconsistent results. While some studies have linked stigma to decreased treatment seeking in Japanese university students, military personnel, and the general public [1, 24], other studies have found that non-stigma barriers such as low perceived treatment need and preferring informal help over treatment may more strongly limit Japanese treatment-seeking willingness [22, 25]. Accordingly, a key goal of this study was to characterize the influence of public stigma and its specific components on treatment-seeking willingness in Japanese adults; providing empirically supported intervention targets to bolster Japanese treatment seeking.

Second, while stigma is often conceptualized and examined as an individual-level construct that affects people’s beliefs, behaviors, and health, we examined public stigma because evidence suggests that people’s stigma beliefs and behaviors are learned from and deeply influenced by the publicly held/enforced stigmatizing beliefs and attitudes of their sociocultural environments [9, 26]. Thus, patterns of public stigma appear to vary widely across cultural groups, with individuals living in environments with strong public stigma (marked by strong prejudicial beliefs and behaviors toward individuals with mental disorders) often avoiding seeking treatment to prevent stigma-related risks of exposure, rejection, and loss of approval/status within their social environments [12].

Third, we studied public stigma because these stigmatizing beliefs and attitudes may carry outsized weight and relevance for treatment seeking in Japan, where: (1) mental illness stigma is heightened, and (2) social approval, conformity, and protection of individual and family status/reputation are culturally prioritized [18, 27]. Consequently, identifying national patterns of public mental illness stigma in Japanese adults may provide useful insights into how culturally held stigma beliefs and attitudes may create a national environment in which mental health treatment seeking is either supported or discouraged to limit exposure to judgment or exclusion [5, 14].

### Public stigma and treatment seeking

To investigate how public stigma may affect treatment seeking among Japanese adults, the current national study examined Japanese adults’ public stigma using established stigma measures to facilitate international comparison. We organized public stigma into two treatment-seeking relevant domains for empirical investigation: (1) public stigma beliefs involving perceived seriousness, treatment and recovery expectations, and neurobiological/sociomoral attributions of mental disorders, and (2) public stigma attitudes involving desired social distancing from mental disorders and perceived dangerousness.

The first domain, public stigma beliefs, captures whether Japanese adults view mental disorders as serious health conditions, believe these conditions can improve with professional treatment or on their own, and attribute these conditions to sociomoral (e.g., bad character, poor upbringing) or neurobiological causes (e.g., genetics, chemical imbalance). This domain is important because low perceived need and low expectations for recovery are known treatment-seeking barriers in Japan [25], and sociomoral attributions have been shown to be more stigmatizing and less supportive of treatment seeking than neurobiological ones [28, 29].

The second domain captures two public stigma attitudes that together have been labeled stigma’s “backbone” [30]. The first, desired social distance, reflects whether people are willing to live near, socialize with, befriend, work with, or have a family member marry someone with a mental disorder while the second, perceived dangerousness, captures whether people believe someone with a mental disorder may be dangerous to themselves or others. These attitudes are important to study as Japanese research has consistently shown high levels of desired social distance by Japanese adults from individuals with mental disorders, which may play a role in limiting treatment-seeking behaviors in this population [18, 31].

Beyond investigating public stigma’s effects on treatment seeking, to boost future intervention efforts, we also examined two potential treatment-seeking facilitators that could be particularly relevant for Japanese adults: prior treatment experience and mattering to others. Prior treatment experience was examined as individuals who have engaged with the Japanese mental health system may have reduced uncertainty, more favorable treatment perceptions, and fewer negative stereotypes of treatment that could weaken public stigma’s impact on treatment-seeking willingness [32]. Prior treatment experience may therefore serve as a key treatment facilitator in Japanese adults by increasing familiarity with professional treatment and strengthening treatment confidence as an acceptable response to mental disorders.

The second facilitator examined was mattering—defined as a person’s perception that they are known, important, and relied upon by others—as mattering has been identified as a core psychological need that may influence mental health by strengthening social belonging and reinforcing the idea that one’s mental health and recovery are important to others [33–35]. Mattering may hold particular salience as a potential treatment facilitator within Japan’s collectivist culture based on empirical evidence that mattering to others: (1) may buffer against stigma’s negative impact on individuals with mental disorders [36], and (2) has been shown to be a potent protective psychological resource against mental health problems such as depression, anxiety, and suicidality in Japanese adults [37]. Yet, despite evidence linking mattering to mental health, stigma, and recovery, its relationship with treatment-seeking willingness remains unclear, supporting the present examination of mattering as a potential treatment-seeking facilitator among Japanese adults.

### Purpose

Because mental illness stigma in Japan is among the world’s strongest but the national patterns and effects of Japanese public stigma on treatment seeking remain unclear, this study pursued three aims. The first aim was to characterize Japan’s public stigma beliefs and attitudes in a national sample of adults and compare it to the beliefs and attitudes of the U.S. public using data from the nationally representative 2018 General Social Survey (GSS) of U.S. stigma [17]. Based on previous findings of elevated mental illness stigma and reduced treatment seeking in Japan relative to other nations [3, 18, 19], we hypothesized that, relative to U.S. adults, Japanese adults would be more likely to: (1) perceive mental disorders as less serious health conditions, (2) have lower expectations of treatment effectiveness and mental health recovery, (3) attribute mental disorders more frequently to sociomoral causes and less frequently to neurobiological causes, (4) desire greater social distance across social situations, and (5) perceive individuals with mental disorders as more dangerous to themselves or others. The second study aim was to examine which components of Japanese public stigma independently predicted treatment-seeking willingness to identify targets for future intervention. We hypothesized that stronger public stigma beliefs and attitudes—particularly lower perceived seriousness and treatment/recovery expectations, and greater social distance—would be associated with lower treatment-seeking willingness in Japanese adults. The third aim was to examine whether the potential facilitators of mattering and prior treatment experience would promote Japanese adults’ treatment-seeking willingness when accounting for public stigma’s effects. We hypothesized that greater mattering and prior treatment experience would each independently predict greater treatment-seeking willingness in Japanese adults.

## Methods

### Study design

Study procedures were approved by the University of California, Riverside Institutional Review Board. Survey data were collected from 1,012 Japanese adults through Qualtrics Research Panels in 2024. Qualtrics recruited participants through multiple panels/digital platforms and has been validated as generating effective demographically representative samples [38], including for previous Japanese validation studies [39] and Japanese health research [40–42]. Geographic and gender-based quota sampling procedures achieved a geographically representative national sample of Japan’s population by region (e.g., Kanto, Kansai, Hokkaido) and gender. The survey was translated into Japanese by professionally-certified translators using a four-stage process involving: (1) primary translation into Japanese from English, (2) secondary translator reviewing the translated survey, (3) consensus-based resolution of differences between the translators, and (4) final editing/review by Japanese research team members.

Participants completed the informed consent and survey through the Qualtrics portal. Adhering to an established data screening protocol [43], Qualtrics applied data quality screening measures that were followed by manual data quality checks by the research team. To compare the public stigma beliefs and attitudes of Japan versus the U.S., the resulting Japanese dataset from 2024 was compared to 2018 U.S. GSS data, which included 480 U.S. adults who completed identical depression or schizophrenia vignettes to this study. The U.S. was selected as the comparison country because the GSS provides nationally representative data using the same established vignette-based public stigma measures administered in the current study [15], enabling direct comparison of Japanese public stigma beliefs and attitudes with one of the most extensively characterized stigma datasets in the research literature [17].

### Measures

Participants provided demographic information including age, gender, and education. Screened major depressive disorder (MDD) and generalized anxiety disorder (GAD) were determined using the summed cutoff scores of 10 + on the widely-used Patient Health Questionnaire-9 (PHQ-9) and Generalized Anxiety Disorder-7 (GAD-7) questionnaires [44, 45]. Cronbach’s alphas for the PHQ-9 and GAD-7 were 0.92 and 0.94, respectively.

Need for past-year mental health treatment was assessed using the commonly used Medical Expenditure Panel Survey item that asked participants, “*In the past 12 months*,* was there a time when you wanted to talk with or seek help about stress*,* depression*,* or problems with emotions?*” Treatment-seeking attitudes and behaviors were measured using adapted items from the National Comorbidity Survey [32, 46] that assessed the following constructs: (1) treatment-seeking willingness (“*If you had a serious emotional problem*,* would you seek professional help?*”), (2) treatment comfort (“*How comfortable would you feel discussing personal problems with a professional?*”), (3) treatment worry (“*How worried would you be that needing help might affect your job prospects?*”), and (4) prior treatment experience (“*Have you ever sought professional help for an emotional or mental health problem?*”). Treatment-seeking willingness served as the primary outcome in regression analyses; treatment comfort, treatment worry, and perceived need were examined descriptively, while prior treatment experience was examined as a potential treatment-seeking facilitator.

Mental illness stigma was assessed via established stigma modules previously used globally [15, 30] and in Japanese stigma research [27, 47, 48]. In our study, Japanese participants were presented with randomly assigned vignettes depicting a gender-matched Japanese man (Kentaro) or woman (Aiko) with either MDD or schizophrenia. Participants were randomly assigned 1:1 by the Qualtrics survey platform to the MDD or schizophrenia vignette, resulting in 505 participants completing the MDD vignette and 507 participants completing the schizophrenia vignette. The random allocation for the U.S. GSS study was also 1:1 with 251 U.S. participants completing the MDD vignette and 259 U.S. participants completing the schizophrenia vignette.

After reading the vignette, participants answered a series of standardized stigma questions assessing the perceived: (1) seriousness of the character’s mental disorder (1 = *not serious at all*, 4 = *very serious*), (2) treatability involving how likely the mental disorder would improve on its own, with professional treatment, and with traditional/cultural treatments (1 *= very unlikely*, 4 *= very likely*), and (3) causal attributions by asking participants to rate the likelihood the character’s mental disorder was caused by various factors **(**e.g., “*a chemical imbalance in the brain*,” “*a genetic or inherited problem*,” “*their own bad character*,” “*the way they were raised*”) (1 *= very unlikely*, 4 *= very likely*). Neurobiological attributions were coded as present if participants rated either “*chemical imbalance*” or “*genetics*” as “*likely*” or “*very likely*.” Sociomoral attributions were coded if participants rated “*bad character*” or “*way raised*” as “*likely*” or “*very likely*.”

Desired social distance was measured using the five-item GSS scale [15] that assessed participants’ willingness to: (1) live next door to, (2) spend an evening with, (3) be friends with, (4) work closely with, and (5) have the character marry into their family (1 *= definitely willing*, 4 *= definitely unwilling*). Responses were averaged to generate a composite five-item social distance score with identical Cronbach’s alphas of 0.88 for the Japanese sample and 0.88 for the U.S. sample. Perceived dangerousness was assessed via two items evaluating the character’s likelihood of violence toward others and toward oneself.

Mattering was assessed for Japanese participants via the six-item General Mattering Scale (GMS-6; [49]), a unidimensional measure of individuals’ perceived significance to others that encompasses feeling important, noticed, cared about, relied upon, missed, and listened to by other people. Sample items include “*How important do you feel you are to other people?”* and “*How much do other people show they care about you?*” (1 *= not at all*, 4 *= a lot*). The scores for the six items are summed to produce a total GMS-6 summed score ranging from 6 to 24 that was used for analyses, with higher scores indicating greater perceived mattering to others. The Cronbach’s alpha for the GMS-6 was 0.94.

### Statistical analysis

Analyses were conducted in STATA v.18.0. Descriptive analyses included frequencies, means, and standard deviations. For ease of interpretation in descriptive analyses, the treatment-seeking and public stigma variables were dichotomized by grouping similar response options (e.g., “*likely*” included “*somewhat likely*” and “*very likely*” responses, “*unwilling*” included “*probably unwilling*” and “*definitely unwilling*” responses), as in previous studies [15, 50]. Dichotomization was applied only to the descriptive and cross-national comparative analyses (Tables 2 and 3), with the binary categories corresponding to substantively meaningful thresholds (e.g., willingness versus unwillingness to interact, endorsement versus non-endorsement of a causal attribution) to maintain comparability with prior GSS-based national stigma estimates reported in this precise dichotomous format [15, 17]. However, the linear regression analysis utilized the original 4-point response scales (e.g., *“very likely”*, *“somewhat likely*”, “*somewhat unlikely*”, “*very unlikely*”), to avoid potential information loss from dichotomization. To compare public stigma variables between the Japanese and U.S. samples, we conducted logistic regressions to estimate predicted probabilities, derived from average marginal effects, for each outcome for the depression and schizophrenia vignettes separately; accounting for unequal sample sizes [51, 52].

**Table 1:** Demographic characteristics for Japanese national sample (N = 1,012)

Variables		% (# of cases)
Gender		
Female		48.1 (487)
Male		51.8 (524)
Education		
< High school		3.4 (34)
High school		28.4 (287)
Vocational/some college		22.4 (227)
≥ College		45.4 (459)
Region		
Kanto		35.3 (357)
Kansai		19.1 (193)
Kyushu/Okinawa		11.5 (116)
Chubu		11.3 (114)
Tohoku		7.3 (74)
Chugoku		6.9 (70)
Hokkaido		4.6 (47)
Shikoku		4.0 (40)
Mental Health		
Fair/poor mental health		55.5 (562)
Screened MDD or GAD		26.3 (266)
Mental Health Treatment		
Treatment-seeking willingness		53.7 (543)
Prior treatment experience		22.5 (228)
Treatment comfort		45.6 (461)
Treatment worry		39.4 (399)
Needed treatment in past year		21.2 (215)
		(Mean ± SD)
Age (Years)		51.9 ± 12.9
Mattering		15.6 ± 5.4

MDD = Major depressive disorder; GAD = Generalized anxiety disorder

**Table 2:** Public stigma beliefs of perceived seriousness, treatability, and causal attributions for 2024 Japanese national sample vs. 2018 GSS U.S. national sample

Perceived Cause	Japan Sample %		U.S. GSS Sample %	
	MDD(n = 505)	Schizophrenia(n = 507)	MDD(n = 251)	Schizophrenia(n = 259)
Treatment				
Serious problem	**89.9**	**91.3**	**95.4**	**97.8**
Improve on own	**25.3**	24.5	**33.6**	18.3
Improve with treatment	**77.8**	**78.7**	**95.4**	**96.4**
Improve with cultural treatment	20.8	24.1	--	--
Neurobiological Attributions				
Chemical imbalance in the brain	**54.1**	**61.1**	**82.2**	**93.5**
Genetic or inherited problem	**29.7**	**34.3**	**66.2**	**76.1**
Neurobiological conception (2-item)	**62.6**	**68.2**	**86.9**	**95.5**
Sociomoral Attributions				
Own bad character	**18.8**	**24.7**	**35.0**	**37.8**
Way person was raised	45.3	45.2	45.2	43.6
Sociomoral conception (2-item)	50.7	53.8	56.0	60.0

GSS = General Social Survey; MDD = Major depressive disorderBoldface indicates the percentage for the group significantly differed from the percentage for the U.S. public (p<0.05)

We then conducted linear regression with willingness to seek treatment (outcome variable) regressed on the following predictor variables: (1) *covariates* of age, gender, education plus vignette and screened MDD or GAD, (2) *public stigma beliefs* of perceived seriousness and treatability, causal attributions of neurobiological and sociomoral conception, (3) *public stigma attitudes* of desired overall social distance and perceived dangerousness toward self and others, and (4) *potential treatment facilitators* of mattering and prior treatment experience. Exploratory regression analyses additionally tested whether mattering moderated the associations for each of the ‘public stigma beliefs’ and ‘public stigma attitudes’ predictors with willingness to seek treatment by adding each of the interaction terms separately to the adjusted primary linear regression model.

## Results

### Demographics and treatment seeking

Table 1 presents demographic and descriptive statistics of Japanese participants. Over half (56%) of Japanese participants had fair/poor mental health although only 21% perceived needing past-year mental health treatment and 23% reported prior treatment experience. Of the 26% of participants screening positive for MDD or GAD, only 49% perceived needing past-year mental health treatment, demonstrating a sizable gap between participants’ actual versus perceived treatment need.

**Table 3:** Public stigma attitudes of desired social distance and perceived dangerousness for 2024 Japanese national sample vs. 2018 GSS U.S. national sample

Vignette: Major Depressive Disorder	Japan Sample		U.S. GSS Sample	
	Mean ± SD	% Unwilling	Mean ± SD	% Unwilling
Social Distance (5-item)	3.04 ± 0.59	**84.8**	2.44 ± 1.56	**27.5**
Unwilling to move next door	3.14 ± 0.68	**86.9**	2.43 ± 1.80	**18.7**
Unwilling to socialize	3.13 ± 0.75	**84.4**	2.30 ± 1.70	**18.9**
Unwilling to make friends	2.76 ± 0.81	**66.7**	2.17 ± 1.65	**12.9**
Unwilling to work closely on a job	2.86 ± 0.79	**73.5**	2.51 ± 1.63	**30.5**
Unwilling to have person marry into family	3.34 ± 0.68	**91.7**	2.80 ± 1.82	**43.2**
	**Mean ± SD**	**% Likely**	**Mean ± SD**	**% Likely**
Danger to others	2.95 ± 0.77	**25.9**	2.61 ± 1.65	**33.3**
Danger to self	2.44 ± 0.83	**55.0**	3.29 ± 1.49	**81.4**
	**Japan Sample**		**U.S. GSS Sample**	
**Vignette: Schizophrenia**	**Mean ± SD**	**% Unwilling**	**Mean ± SD**	**% Unwilling**
Social Distance (5-item)	3.13 ± 0.62	**86.6**	2.91 ± 1.16	**59.8**
Unwilling to move next door	3.19 ± 0.69	**88.6**	2.90 ± 1.62	**48.1**
Unwilling to socialize	3.22 ± 0.71	**86.6**	2.74 ± 1.41	**45.7**
Unwilling to make friends	2.92 ± 0.79	**73.6**	2.55 ± 1.41	**34.8**
Unwilling to work closely on a job	2.94 ± 0.80	73.2	3.07 ± 1.30	68.3
Unwilling to have person marry into family	3.37 ± 0.70	**92.3**	3.28 ± 1.46	**70.5**
	**Mean ± SD**	**% Likely**	**Mean ± SD**	**% Likely**
Danger to others	2.58 ± 0.82	**48.1**	3.15 ± 1.50	**69.3**
Danger to self	2.24 ±0.89	**64.9**	3.64± 1.36	**91.6**

Note: Percentages represent combined “definitely unwilling” and “probably unwilling” or “very likely” and “somewhat likely” responses GSS = General Social Survey Boldface indicates the percentage for the group significantly differed from the percentage for the U.S. public (p<0.05)

Fifty-four percent of Japanese participants reported willingness to seek treatment for a serious emotional problem, but only 46% felt comfortable talking to a mental health professional and 39% worried treatment would hurt their job opportunities. Significantly more women than men reported needing past-year treatment (25% vs. 17%; *χ*^*2*^(1, *N* = 1011) = 9.42, *p* < 0.01) although significantly fewer women felt comfortable seeking treatment versus men (42% vs. 49%; *χ*^*2*^(1, *N* = 1011) = 4.94, *p* < 0.05).

### Public stigma

For the public stigma beliefs variables in Table 2, compared to the U.S. public, Japanese participants had significantly lower rates of: (1) perceiving MDD and schizophrenia as serious problems (*p* < 0.05), (2) believing MDD and schizophrenia would improve with treatment (*p* < 0.05), and (3) believing MDD would improve on their own (*p* < 0.05). Approximately one quarter of Japanese participants also believed that informal cultural treatments would improve MDD and schizophrenia. For causal attributions, Japanese participants reported significantly lower rates of endorsing neurobiological—but not sociomoral—explanations for MDD and schizophrenia than the U.S. public (*p* < 0.05).

Table 3 reports data on the public stigma attitude variables of desired social distance and perceived dangerousness. Japanese participants reported significantly higher rates of overall desired social distancing than the U.S. public (*p* < 0.05) for both MDD (85% versus 28%) and schizophrenia (87% versus 60%). Relative to the U.S. public, Japanese participants reported significantly higher rates of unwillingness (*p* < 0.05) to interact with individuals with MDD in every social situation including: (1) move next door to, (2) socialize, (3) make friends with, (4) work closely on a job, and (5) have them marry into their family. Toward individuals with schizophrenia, Japanese participants reported significantly higher rates of unwillingness (*p* < 0.05) to: (1) move next door to, (2) socialize, (3) make friends with, and (4) have them marry into their family.

For perceived dangerousness, Japanese participants reported that: (1) individuals with MDD were less likely to be dangerous to themselves or others than individuals with schizophrenia, and (2) individuals with MDD or schizophrenia were more likely to be dangerous to themselves than others. For all perceived dangerousness variables, Japanese participants were significantly less likely to perceive individuals with MDD or schizophrenia as dangerous to themselves or others than the U.S. public (*p* < 0.05).

### Linear regression

Table 4 regression data indicated that of the covariates, age, gender, vignette, and screened MDD or GAD did not significantly predict treatment-seeking willingness while greater levels of education involving graduating from high school (*β* = 0.16, *p* < 0.05) and college (*β* = 0.20, *p* < 0.01) independently predicted greater treatment-seeking willingness. For the public stigma beliefs and attitudes variables, perceiving mental disorders as likely to improve with treatment independently predicted greater treatment-seeking willingness (*β* = 0.18, *p* < 0.01) while perceiving mental disorders as likely to improve on their own predicted lower treatment-seeking willingness (*β* = -0.11, *p* < 0.01). Desiring greater overall social distance independently predicted lower treatment-seeking willingness (*β* = -0.09, *p* < 0.01) while neurobiological or sociomoral attributions and perceived dangerousness to self or others did not independently predict treatment-seeking willingness. For potential treatment facilitators, possessing greater mattering (*β* = 0.22, *p* < 0.01) and prior treatment experience (*β* = 0.28, *p* < 0.01) both independently predicted greater treatment-seeking willingness after controlling for public stigma variables and covariates. Exploratory moderation analyses found no significant interactions between mattering and any public stigma belief or attitude in predicting treatment-seeking willingness, including perceived seriousness, expectations of improvement with or without treatment, neurobiological or sociomoral attributions, desired social distance, or perceived dangerousness to self or others (*β*s = − 0.10 to 0.03, *p*s = 0.41–0.97). Thus, mattering independently predicted greater treatment-seeking willingness but did not significantly moderate the associations between the examined public stigma predictors and treatment-seeking willingness.

**Table 4:** Japanese mental health treatment-seeking willingness regressed on covariates, public stigma beliefs, public stigma attitudes, and treatment-seeking facilitators

DV = Mental Health Treatment Willingness					
Step	Predictor	*β*	*b* (*SE*)	*F*	*R*^2^ (*∆R*^*2*^)
1: *Covariates*	Age	0.04	0.01 (0.01)	2.49^*^	0.01^*^
	Gender	0.02	0.04 (0.06)		
	High school graduate	**0.16** ^*^	**0.33 (0.14)** ^*^		
	Some college	0.12	0.27 (0.14)		
	College graduate	**0.20** ^**^	**0.37 (0.14)** ^**^		
	Vignette completed	− 0.01	− 0.02 (0.05)		
	Screened MDD or GAD	− 0.04	− 0.12 (0.04)		
2: *Public Stigma Beliefs*	Serious problem	0.01	0.01 (0.04)	10.20^**^	0.10^**^ (0.09)^**^
	Improve on own	**− 0.11** ^**^	**− 0.12 (0.04)** ^**^		
	Improve with treatment	**0.18** ^**^	**0.22 (0.04)** ^**^		
	Neurobiological conception	0.02	0.04 (0.06)		
	Sociomoral conception	− 0.01	− 0.02 (0.06)		
3: *Public Stigma Attitudes*	Desired social distance	**− 0.09** ^**^	**− 0.14 (0.05)** ^**^	9.81^**^	0.12^**^ (0.02)^**^
	Danger to others	0.03	0.04 (0.04)		
	Danger to self	0.07	0.07 (0.04)		
4: *Treatment-Seeking Facilitators*	Mattering	**0.22** ^**^	**0.04 (0.01)** ^**^	18.10^**^	0.22^**^ (0.10)^**^
	Prior treatment experience	**0.28** ^**^	**0.62 (0.07)** ^**^		

^*^p < 0.05

^**^p < 0.01

MDD = Major depressive disorder; GAD = Generalized anxiety disorder

## Discussion

Study findings revealed strong Japanese public mental illness stigma beliefs and attitudes that may limit Japanese adults’ willingness to seek mental health treatment. Extending previous Japanese research that documented low adult treatment utilization [1, 4], Japanese participants reported low treatment-seeking willingness, limited comfort engaging with treatment professionals, and low perceived treatment need even among participants with major depressive or anxiety disorders. Only 54% of adults expressed willingness to seek treatment for a serious emotional problem, markedly below the 70% willingness rate for U.S. adults [12].

Among Japanese adults, public stigma was elevated across multiple stigma domains relative to the U.S. in ways likely to be unfavorable to treatment seeking. Interestingly, this elevation was not universal but was concentrated in specific stigma domains. Specifically, Japanese adults reported greater social avoidance-rejection and lower treatment/recovery expectations than U.S. adults, while endorsing dangerousness stereotypes less frequently. Importantly, although public stigma was measured through individual beliefs and attitudes, participants’ responses should not be viewed as functioning independently of the broader social environment. Public stigma may reflect exposure to culturally and historically patterned representations of mental illness, institutional practices, social norms, and anticipated social or reputational consequences [14, 15]. Thus, the stigma beliefs and attitudes identified in this study may represent individual-level manifestations of broader social processes. Yet, because these societal mechanisms were not directly measured and therefore cannot be established by the present analyses, further research is needed to examine how broader Japanese social and institutional contexts shape public stigma.

With regard to specific public stigma domains, the first finding was that Japanese participants reported exceptionally high rates of desired social distancing from individuals with mental disorders with 85% of Japanese participants desiring social distance from individuals with MDD versus only 28% of U.S. adults; exceeding even the social distancing rate U.S. adults desired from individuals with schizophrenia by 25 percent. Although not within the scope of this study to confirm, it is possible that this strong desire by Japanese participants to socially avoid individuals with MDD or schizophrenia may reflect the boundaries Japanese culture draws between one’s inner circle (*uchi*) and outsiders (*soto*), whereby admitting someone with a mental disorder into the *uchi* may be viewed as risking both the individual’s standing and the standing of others within the *uchi* by threatening their collective face (*mentsu*) [53–55]. As a result, desiring social distance from individuals with mental disorders may represent less of a moral judgment against the individual than a protective cultural strategy to shield one’s *uchi* from the reputational costs of stigmatizing associations. In this way, this core Japanese stigma component may also suppress treatment seeking by making salient the high reputational costs/risks associated with being publicly connected to mental illness or its treatment [25] in a cultural context that prioritizes conformity, harmony, and protecting social standing [18]. According to our linear regression data, this possibility was supported as greater social distancing did independently predict lower treatment-seeking willingness among Japanese participants, even after controlling for other factors including demographics and the other public stigma domains.

Second, Japanese adults were significantly less likely than U.S. adults to view mental disorders as serious or believe they would improve with treatment or on their own. Descriptively, this pattern aligns with reports that Japanese mental disorders are expected to be managed privately/on their own [18, 25], and is consistent with survey findings that many Japanese adults are pessimistic about the prospects of full recovery from mental disorders [22, 48]. Based on prior Japanese research, low recovery expectations may therefore reflect both historical and contemporary features of Japan’s mental health system and social context. For example, Japan’s longstanding reliance on long-term psychiatric institutionalization may have limited public visibility of mental illness recovery [18, 22, 56], although more recent reforms have increasingly emphasized community-based care, social integration, and deinstitutionalization [57, 58]. Thus, current low recovery expectations should not be attributed solely to Japan’s institutional history but may reflect the interaction of older treatment models with ongoing changes in mental health treatment and public exposure to recovery.

Countering our study hypotheses, Japanese adults less frequently attributed mental disorders to neurobiological causes, which research has often associated with lower blame than sociomoral explanations [28, 29], while endorsement of sociomoral causes did not significantly differ from the U.S. public. Descriptively, these findings indicate lower endorsement of biomedical explanations among Japanese versus U.S. respondents without evidence of greater rates of sociomoral attribution. However, neurobiological explanations may not be uniformly destigmatizing across cultural contexts. In East Asian settings, beliefs that mental illness is inherited may intensify stigma by implicating family lineage, marriageability, or the reputation of relatives through concerns such as a “tainted bloodline” narrative [59, 60]. Accordingly, increasing Japanese adults’ biomedical understanding should not be assumed to automatically reduce Japanese public stigma. Mental health education/literacy efforts in Japan may therefore consider pairing biomedical explanations of mental disorders with efforts to address any potential misconceptions about neurobiological attributions that may exacerbate rather than alleviate stigma.

Also contradicting study hypotheses, although dangerousness is considered part of stigma’s backbone in Western research [30], perceived dangerousness rates were lower among Japanese adults than the U.S. public, and did not predict treatment-seeking willingness in analyses. These findings suggest that dangerousness is less prominent than social distance and treatment/recovery beliefs among Japanese adults, although our cross-national design could not establish that this reflects culturally-specific differences between Japanese and U.S. adults. Therefore, future research should explore the possibility that dangerousness may not be a dominant stereotype through which public stigma is expressed in Japan, which if confirmed may reflect several factors including Japan’s historic institutionalization/invisibility of individuals with mental disorders creating lower public exposure to dangerousness stereotypes [18] and the Japanese media’s framing of mental disorders in relation to stress and overwork versus dangerousness/violence [61]. Taken together, this finding may support the broader pattern in our data that Japanese public stigma and its effects on treatment seeking may operate through relational/social concerns and disruption to personal and family status, reputation, and roles, rather than the explicit fear-based dangerousness stereotypes that anchor stigma in the U.S.P [17, 30].

Promisingly, as hypothesized, prior treatment experience and mattering to others emerged as potential facilitators for treatment-seeking willingness in Japanese adults, even after accounting for public stigma’s effects. First, prior treatment experience independently predicted greater treatment-seeking willingness, which may suggest that having direct experience with mental health treatment may reduce treatment uncertainty and negative expectations, and make treatment use feel more feasible and acceptable. Although causal conclusions cannot be drawn, these findings suggest that facilitating initial access to positive treatment experiences may increase subsequent openness to seeking professional treatment in response to future mental disorders. Prior treatment experience may therefore represent a useful facilitator for overcoming barriers to treatment seeking among Japanese adults. The second potential facilitator, mattering, also appeared to predict treatment-seeking willingness. Possible theories include that mattering may potentially connect treatment seeking to one’s cultural obligations/responsibilities to one’s self, family, and community [35, 62] in a manner consistent with Japanese collectivistic cultural values [63]. For instance, because interdependent self-construal in Japan emphasizes defining oneself through significant relationships and fulfilling social roles [64–66], mattering may bolster treatment seeking in Japanese adults by reframing treatment seeking as socially consequential to preserving one’s ability to fulfill valued social roles and responsibilities to the family, coworkers, and community [67, 68]. However, exploratory moderation analyses found no evidence that mattering altered the associations between any measured public stigma component and treatment-seeking willingness, suggesting that mattering may operate as an independent treatment-seeking predictor rather than by buffering public stigma’s effects. Consequently, mattering may represent a potential intervention target for increasing treatment seeking among Japanese adults [37], warranting further investigation of this relationship and its possible mechanisms.

The study findings also suggest that public stigma-related treatment barriers are unlikely to be adequately addressed through changes in individual knowledge or beliefs alone. Given that public stigma is often influenced/sustained by broader social norms and institutional environments [9, 14, 15], addressing its effects may require multilevel approaches that combine population-level mental health education and contact-based stigma reduction with institutional and community efforts to normalize treatment/recovery and reduce the social and reputational consequences associated with mental illness and its treatment in Japan. Positively, these multilevel approaches may be beneficial by aligning potential intervention strategies more closely with the broader social processes that may sustain the public stigma beliefs and attitudes observed in this study.

Collectively, our study findings also carry direct implications for mental health equity in Japan. For example, despite universal health insurance coverage and the availability of mental health services [69, 70], our data suggest that public stigma may create barriers that inhibit adults with mental disorders—who may face elevated stigma risk/threats from their social environment—from seeking treatment, as reflected in only 21% of Japanese participants perceiving a need for past-year treatment despite 56% reporting fair/poor mental health. In this way, public stigma may serve as a social determinant of treatment inequity in Japan rather than solely an individual-centered phenomenon. The gender pattern observed in our data may also reflect a potential treatment inequity, as women reported significantly greater perceived treatment need than men yet significantly lower comfort discussing personal problems with a professional. This disparity may partially reflect gendered differences in permitted/preferred sources of support, as prior research has found that Japanese women report stronger intentions and attitudes than men to seek help from informal sources (e.g., family, friends) over professional treatment (e.g., psychologists, physicians) [71]. Notably, because public stigma is amenable to population-level intervention through anti-stigma campaigns and contact-based programs [72, 73], these findings also identify actionable targets for narrowing Japan’s treatment gap and advancing more equitable mental health treatment seeking.

Several study limitations are noted. First, treatment seeking was assessed through treatment willingness, comfort, perceived need, and prior experience. Future studies should link these factors to actual treatment utilization over time due to potential divergences between treatment intentions and utilization (e.g., due to structural barriers such as lack of access to mental health providers). Second, Japanese participants exhibited smaller standard deviations than U.S. participants across multiple stigma measures, which may partly reflect culturally patterned response styles such as response clustering, midpoint or acquiescent responding, or social desirability rather than substantive differences in stigma beliefs and attitudes. Third, differences between the Japanese participant and U.S. comparison samples in demographic composition, recruitment, survey administration, and data collection prevent attributing some observed differences solely to cross-national differences. This includes differences in the data collection timeframes that may have led to observed differences due to temporal changes (e.g., the COVID-19 pandemic). Further, as measurement invariance between the Japanese and U.S. instruments was not tested, group differences should thus be interpreted as descriptive rather than validated cultural differences. However, despite these limitations, this study is significant as it revealed key Japanese public stigma characteristics and predictors of willingness to seek treatment, contributing to an improved understanding of stigma in Japan and possible areas of intervention to increase low treatment-seeking rates in Japanese adults.

## Conclusion

Overall, study results are descriptively consistent with a pattern of strong Japanese public stigma marked by high social distancing, low treatment/recovery beliefs, and low perceived seriousness that may inhibit treatment seeking in Japanese adults. Although the cross-national comparisons should be interpreted cautiously in the absence of additional confirmatory evidence, the within-Japan findings suggest that stigma may be characterized by greater desired social distancing and treatment/recovery pessimism rather than perceived threat/dangerousness stereotypes; highlighting these stigma components as potentially relevant intervention targets for Japanese adults. Mattering emerged as a possible treatment-seeking facilitator that potentially aligns with Japanese cultural values, along with prior treatment experience. Consequently, our findings suggest that treatment seeking in Japan may be strengthened through interventions that integrate stigma reduction and mattering—connecting treatment seeking to recovery, social belonging, and responsibilities to others to reduce unmet treatment need and promote mental health equity in Japan.

## Data Availability

Study data and materials may be made available upon request of the study authors.

## Abbreviations

GAD: Generalized anxiety disorder
GSS: General social survey
MDD: Major depressive disorder
U.S.: United States
